# Undernutrition and associated risk factors among school age children in Addis Ababa, Ethiopia

**DOI:** 10.1186/s12889-015-1714-5

**Published:** 2015-04-12

**Authors:** Dawit Degarege, Abraham Degarege, Abebe Animut

**Affiliations:** Lia Foundation, 1695, Addis Ababa, Ethiopia; Aklilu Lemma Institute of Pathobiology, Addis Ababa University, 1176, Addis Ababa, Ethiopia; Department of Epidemiology, Robert Stempel College of Public Health and Social Work, Florida International University, Miami, FL USA

**Keywords:** Undernutrition, Stunting, Underweight, Risk factors, Children, Addis Ababa, Ethiopia

## Abstract

**Background:**

Causes of child undernutrition are diverse and change in space and time. Investigating current determinants of undernutrition remains vital to design an effective intervention strategy. The study assessed prevalence of undernutrition and its associated factors among children living in Addis Ababa, Ethiopia.

**Methods:**

A community based cross-sectional study was conducted in 459 school age children and their parents or caregivers living in Lideta sub-city, Addis Ababa, Ethiopia. Participants were selected using a multi-stage simple random sampling technique. Height and weight of children was measured and their parents or care givers were interviewed for factors associated with undernutrition.

**Results:**

About 31% (n = 141) of the children were undernourished (19.6% stunted, 15.9% underweight). Being male, higher birth order (>2), larger family size (6–8), low meal frequency (≤3 times) in a day prior to the survey and mud floor house were significantly associated with undernutrition. Similarly, the risk of underweight increased significantly with an increase in age, birth order, family size and also the absence of hand washing facilities. The odds of undernutrition was lower in children born to 20–30 years old mothers compared to those born to mothers younger than 20 years.

**Conclusions:**

Undernutrition is prevalent among school age children living in Lideta sub city, Addis Ababa. Policy makers should consider school age children in their nutrition policy documents and implement screening program and intervention strategy.

## Background

Undernutrition contributes to half of all deaths and 28% of stunting in children worldwide [1]. In the developing countries, 52% and 34-62% of the school-age children are stunted and underweight, respectively [1,2]. If interventions are not carried out, it is estimated that close to one billion children will be physically and mentally impaired by 2020 [3,4]. As school age is a period of physical and mental development, prolonged undernutrition in this age group impairs their growth [5]. Undernutrition could also increase the susceptibility of children to disease [6]. Despite continued prevention efforts, child undernutrition remains a major public health problem in Sub Saharan Africa, including Ethiopia [1,2,7,8].

Factors including biology, economy, culture, environment and disease contribute to undernutrition [9]. Children are most vulnerable to undernutrition due to their low dietary intake, less access to food, inequitable distribution of food within households, improper food storage and preparation, dietary taboos and infections with pathogens [1]. Child undernutrition can be mitigated through nutritional information campaigns, broader access to maternal and child health care practices and availing affordable, diverse, and nutrient-rich food [9].

The Federal Government of Ethiopia has been working to reduce undernutrition significantly through public education and providing nutritional supplements and financial support to vulnerable families. However, the risk factors of undernutrion are diverse and could potentially change in space and time. Thus, there is a need to determine the current nutritional status to review the pitfalls and design effective intervention strategies. This study described the prevalence of undernutrition and factors associated with the problem among school children living in Lideta Sub-City, Addis Ababa.

## Methods

### Study area and design

A community based cross-sectional study was conducted among school age children living in *Woreda* (District) 8, Lideta Sub-City, Addis Ababa, Ethiopia, in March 2014. Lideta Sub-City is one of the ten Sub-Cities in the city. Woreda 8 is one of the ten woredas in the Sub-City and comprises two *Kebeles* (villages), with a total population of 6,163. The woreda has two government hospitals and two health centers, in addition to private clinics. It also has one vocational and technical school, four primary schools and a kindergarten. Most of the adult males living in the woreda are employed or merchants while most adult females mothers are housewives. Members of the community differ in their ethnic background. School age children (5 to 14 years old) who lived in the sub city for at least 5 years and consented through their mothers or caregivers, participated in the study. Severely ill (i.e. not able to communicate or provide the required information due to illness) children were excluded from the study.

### Data collection procedures

Based on 24% prevalence [10], 5% significance level (alpha), 1.5 design effect and 10% non-response rate [11], the sample size was estimated to be 462. This size was divided proportionally between the two *Kebeles*. The number of households in each *Kebele* was then divided by the respective sample size to determine the interval between consecutive study houses. Anthropometric measurements were made for each participating child. When the number of children in a house was greater than one, a child was randomly selected using lottery method. In the absence of an eligible child in the selected household, the next house was considered.

The mother/guardian or caregiver of each participating child was interviewed for potential determinants of child nutrition status, including socio-economy, demography, environment, health related issues and age of the child using a pre-tested structured questioner. The questionnaire was first developed in English and then translated to the Amharic language. Personal hygiene (e.g. presence or absence of dirt in finger nails, frequency of bathing in a week, occasions of hand washing, cleanness of hair, cleanness of clothes) of the child and cleanliness (e.g. environmental and sanitary facilities) of the child’s house were observed and documented using a checklist of items. Three medical nurses, having previous experience in similar data collection, participated in the survey.

### Data quality control

Data collectors were trained in relation to the study for one day. The questionnaire was pre-tested in an area where the study was not undertaken and errors were corrected accordingly. The weight of each child was measured using a calibrated digital balance. Each day, collected information was reviewed and errors were returned to data collectors for correction. Data validity and reliability was maintained through close supervision of data collectors by the first author.

### Anthropometric measurement and nutritional status assessment

Each child was instructed to stand on the center of a digital balance. The digital balance had a vertical wooden bar with plastic tap attached to it. Weight and height of each child was measured after calibrating to the nearest 0.1 kg and 0.1 cm, respectively. Height was measured against a scale where a flat head piece (attached to the plastic tap at a right angle) touched the crown of the head and formed a right angle. Each child was measured while wearing light clothes after removing shoes, belt, cap or any other material that could interfere with their actual height and weight. *Z*-score was determined using the Anthro-Plus software [12]. Based on the *Z*-score, each child was grouped as undernourished or not undernourished [13]. A child was considered undernourished when either underweight (weight-for-age *Z* score or body mass index for-age *Z* score < −2) or stunted (height-for-age *Z* score < −2).

### Data analysis

Data were entered into excel sheet and analyzed using STATA version 11. Frequency distribution tables were used to describe socio-demographic and nutritional status of the study participants. Bivariate and multivariate logistic regression analyses were used to quantify the magnitude of association between different factors and undernutrition. The explanatory variables included in the multivariate regression model were: I. Socio-economic and demographic characteristics of parents [age, sex, family size, parents’ average monthly income, education, occupation, religion and family decision maker], II. Child characteristics and health care practices [age, sex, illness/infection status (ill or not), personal hygiene (presence or absence of dirt in children’s finger nails, frequency of child bathing in a week, occasions of hand washing, cleanness of hair, cleanness of clothes), dietary intake (daily meal frequency), birth order, immunization status (immunized: yes, no), number of general breast milk feeding years], III. Environmental characteristics [source of drinking water, housing conditions (presence or absence of window, nature and cleanness of the floor), availability and cleanness of sanitary facilities such as latrine, hand washing facilities near the toilet, bathing rooms, garbage disposals]. The outcome variables included in the regression model were stunting, underweight and undernutrition. A p-value less than 0.05 was considered as statistically significant.

### Ethical considerations

The study protocol was reviewed and approved by the Institutional Research Review Committee of the College of Medicine and Public Health, Debre Markos University. The objectives and protocol were discussed with the community leaders for clarification. Participation in the study was on a voluntary basis. Oral informed consent was obtained from every participant and his/her parent before conducting the survey. Privacy and confidentiality of the information was ensured. Severely malnourished children were referred to the nearest health facility and advice was given to their parents.

## Results

### Socio demographic characteristics

A total of 459 children (55.99% male) and their parents or caregivers were enrolled in the study (Tables 1 and 2). Most (98%) of the children were breastfed, among whom 14.6% and 43.8% were fed for less than 12 months and 12–24 months, respectively. Close to half of the children (49.0%) had daily meals of greater than three times a day prior to the survey. About 61.7% and 38.3% of the children had a history of illness/infection in the year and in the two weeks prior to the study period, respectively.

**Table 1:** **Socio demographic characteristics of children and their families in Woreda 8, Lideta Sub-City, Addis Ababa, Ethiopia, March 2014**

**Variable**	**Categories**	**Number of observations**	**Relative frequency**
Marital status	Married	369	80.4
	Divorced	33	7.2
	Widowed	51	11.1
	Unmarried	6	1.3
Family size	2-5	307	66.9
	6-8	134	29.2
	>8	18	3.9
Ethnicity of mother/caregiver	Oromo	135	29.4
	Amhara	129	28.1
	Tigre	84	18.3
	Gurage	81	17.7
	Other	30	6.5
Religion of mother/caregiver	Orthodox	318	69.9
	Muslim	75	16.3
	Protestant	36	7.8
	Catholic	27	5.9
	Others	3	0.7
Educational status of mother/caregiver	Cannot read and write	51	11.1
	Can read and write only	28	6.1
	Primary education	191	41.6
	Secondary education	151	32.9
	College and above	33	7.2
	Other	5	1.1
Monthly income of the family (in Ethiopian Birr)	<1000	54	11.8
	1000-2000	309	67.3
	>2000	96	20.9
Maternal age	20-35	267	58.9
	36-45	147	32.5
	>45	39	8.6
Maternal age at child birth	<20	78	16.9
	20-30	321	69.9
	>30	60	13.1
Age of children in years	5-9	177	38.6
	10-14	282	61.4
Sex of children	Male	257	55.9
	Female	202	44.0
Working status of mother	House wife	185	41.0
	Employed	266	59.0

Can read and write only*: individuals who attended non-formal education.

**Table 2:** **Child, maternal and environmental characteristics of in Woreda 8, Lideta Sub-City, Addis Ababa, Ethiopia, March 2014**

**Variable**	**Category**	**Number of observations**	**Relative frequency**
Frequency of infection/ illness in a year prior to the study period⁯	No illness	102	22.2
	1	114	24.8
	>1	243	52.9
Presence of illness/infection in the last two week	Yes	176	38.3
	No	283	61.7
Nature of the floor of the house	Cement/brick/ceramic	351	76.5
	Soil	108	23.5
Presence of dirt in finger nails	Yes	216	47.1
	No	243	52.9
Meal frequency in a day prior to data collection	>3	225	49.0
	3	177	38.6
	<3	57	12.4
Main source of power used for cooking	Electric	332	72.3
	Wood/kerosene	127	27.7
Duration of general breast feed(in months)	Not breast feed	10	2.2
	<12	67	14.6
	12-24	201	43.8
	>24	12	6.4
	Do not remember	60	13.1
Birth order	≤2	321	69.9
	>2	138	30.1
Bath taking of the child	Daily	18	3.9
	2-4 times/week	48	10.5
	Weekly	393	85.6
Presence of hand washing facility near the latrine	Yes	177	39.6
	No	270	60.4
Main decision maker of the household	Father	204	44.7
	Mother	82	17.9
	Both by discussion	138	30.1
	Not sure	35	7.4

⁯Illness/Infection: illnesses that enforces children to visit health centers/hospitals and take medication.

### Undernutrition and its risk factors in school age children

Out of the 459 children, 30.9% were undernourished (stunted = 19.6%, underweight = 15.9%). Having a birth order greater than two, meal frequency at most three times a day, large family size (6–8), being born to a mother less than 20 years old, living in mud/soil floored house and being male were significantly associated with increased odds of undernutrition in the multivariate regression model (Table 3).

**Table 3:** **Factors associated with undernutrition among school age children in Woreda 8, Lideta Sub-City, Addis Ababa, March, 2014**

**Variable**	**Categories**	**Undernutrition**		**Crude odds ratio (95% CI)**	**Adjusted odds** ^**¶**^ **ratio (95% CI)**
		**Yes**	**No**		
Ethnicity	Oromo	54	81	1	1
	Amhara	29	100	0.44 (0.25-0.74)**	1.13 (0.39-3.27)
	Tigre	24	60	0.60 (0.33-1.08)	0.19 (0.06-0.52)
	Gurage	25	56	0.67 (0.37-1.20)	0.02 (0.01-0.17)
Religion	Orthodox	93	225	1	1
	Muslim	18	57	0.76 (0.43-1.38)	0.60 (0.21-1.73)
	Protestant/Catholic or others	30	36	2.01 (1.17-3.46)*	1.75 (0.74-4.18)
Family size	2-5	100	207	1	1
	6-8	41	93	0.91 (0.58-1.41)	3.23 (1.43-7.29)**
	>8	7	18	0.81 (0.34-6.52)	2.13 (0.97-8.91)
Child age	5-9	39	138	1	1
	10-14	102	180	2.00(1.30-3.08)**	1.31(0.65-2.66)
Child Sex	Male	88	169	1	1
	Female	53	149	0.68 (0.46-1.02)	0.44 (0.20-0.93)*
Maternal age at child birth	<20	40	38	1	1
	20-30	77	244	0.29 (0.18-0.50)***	0.14 (0.06-0.51)***
	>30	24	36	0.63 (0.32-1.25)	0.24 (0.18-1.18)
Floor of the house	Cement	96	255	1	1
	Soil	45	63	1.89 (1.21-2.97)*	2.43 (1.05-5.59)*
Source of power for cooking	Electric	51	54	1	1
	Wood/Kerosene	90	264	0.36 (0.23-0.57)***	0.46 (0.20-1.08)
Meal frequency in a day	>3	56	169	1	1
	≤3	85	149	1.72 (1.15-2.58)**	1.77 (1.23-2.55)**
Birth order	≤2	84	237	1	1
	>2	57	81	1.99 (1.30-3.02)**	2.14 (1.27-3.59)**

Note: * = P < 0.05, ** = P < 0.01, *** = P <0.001.

**¶** ^**=**^adjusted (from multivariate logistic regression model) for socio-economic and demographic characters of parents and children and health caring practices and environmental conditions (detail list available in the data analysis section), but only values of variables which showed significant association either in the bivariate or multivariate regression analysis were reported in the table.

### Factors associated with child stunting

The odds of stunting was significantly high in children living in a house having mud floor, birth order of >2, had at most 3 times daily meals, having employed and protestant or catholic mother (Table 3). Children born to mothers less than 20 years of age at the time of birth were associated with an increased risk of stunting (Table 4).

**Table 4:** **Factors associated with stunting in school age children in Woreda 8 of Lideta sub-city, Addis Ababa, March, 2014**

**Variable**	**Categories**	**Stunting**		**Crude odds ratio (95% CI)**	**Adjusted odds ratio** ^**¶**^ **(95% CI)**
		**Yes**	**No**		
Religion	Orthodox	54	264	1	1
	Muslim	12	63	0.93 (0.47-1.84)	1.25 (0.42-3.69)
	Protestant/Catholic	24	42	2.79 (1.56-4.99)**	3.29 (1.15-9.39)*
Maternal educational status	No formal education	18	61	1	1
	Primary	19	172	0.37 (0.18-0 .76)**	1.99 (0.12-3.26)
	Secondary and above	53	131	1.37 (0.74-2.54)	6.96 (2.94-16.46)
Frequency of illness/infection in a year prior to the study period⁯	No infection	12	90	1	1
	1	18	96	1.41 (0.64-3.08)	0.07 (0.01-0.46)
	>1	60	183	2.46 (1.26-4.80)**	1.34 (0.35-5.09)
Presence of dirt in finger	Yes	55	161	1	1
	No	35	208	0.49 (0.31-0.79)**	0.2 (0.37-1.18)
Floor material of the house	Ceramic	48	303	1	1
	Mud	42	66	4.02 (2.46-6.57)	7.94 (2.22, 28.36)***
Birth order	≤2	51	270	1	1
	>2	39	99	2.09 (1.29-3.36)*	5.43 (2.62-11.25)***
Maternal age	20-35	39	228	1	1
	36-45	45	102	2.58 (1.58-4.20)***	4.43 (2.08-9.47)***
	>45	8	37	1.26 (0.40-2.70)	1.72 (0.54-5.50)
Maternal age at child birth	<20	19	59	1	1
	20-30	50	271	0.57 (0.31-1.04)	0.09 (0.03-0.29)***
	>30	21	39	1.67 (0.79-3.51)	0.24 (0.04-1.40)
Working status of mother	House wife	28	157	1	1
	Employed	62	204	1.70 (1.04-2.79)*	1.89 (1.38-5.03)*
Meal frequency in a day	>3	26	199	1	1
	≤3	64	170	2.88 (1.75-4.75)***	4.62 (2.71-7.89)***

Note: * = P < 0.05, ** = P < 0.01, *** = P <0.001.

**¶** ^**=**^adjusted (from multivariate logistic regression model) for socio-economic and demographic characters of parents and children and health caring practices and environmental conditions (detail list available in the data analysis section), but only values of variables which showed significant association either in the bivariate or multivariate regression analysis were reported in the table.

⁯Illness/Infection: illnesses that enforces children to visit health centers/hospitals and take medication.

### Factors associated with child underweight

Information about factors associated with children being underweight is summarized in Table 5. The likelihood of being underweight was significantly higher among children 10–14 years old than among children 5–9 years old. Children with families of 6–8 members and having no hand washing facility were more likely to be underweight compared to those with less than 6 members and having said washing facility, respectively.

**Table 5:** **Factors associated with underweight among school age children in district 8, Lideta sub-city, Addis Ababa, Ethiopia, March, 2014**

**Variable**	**Category**	**Underweight**		**Crude odds ratio (95%CI)**	**Adjusted odds ratio** ^**¶**^ **(95%CI)**
		**Yes**	**No**		
Family size	2-5	43	264	1	1
	6-8	29	105	1.65 (0.98-2.78)	2.82 (1.45-5.48)**
	>8	0	18	NA	NA
Main decision maker	Father	49	155	1	1
	Mother	10	72	0.44 (0.21-0.92)*	0.39 (0.16-0.94)*
	Both by discussion	14	159	0.28 (0.14-0.59)***	0.11 (0.04-0.26)***
Ethnicity of mother	Oromo	31	104	1	1
	Amhara	11	118	0.31(0.15-0.65)**	0.32 (0.13-0.79)*
	Tigri	21	63	0.12(0.59-2.11)	1.72 (0.77-3.74)
	Gurage	4	77	0.17(0.59-0.51)**	0.12 (0.04-0.42)**
	Others	6	24	0.84 (0.47-1.43)	0.29 (0.08-1.12)
Maternal age	20-35	49	221	1	1
	>35	25	164	0.69 (0.43-1.27)	0.38 (0.15-0.97)*
Maternal age at birth	<20	25	53	1	1
	≥20	47	334	0.31 (0.11-0.39)***	0.35 (0.15-0.81)*
Birth order	≤2	59	238	1	1
	>2	39	123	1.28 (0.74-1.98)	2.85 (1.35-6.02)**
Presence of hand washing facility	Yes	39	240	1	1
	No	35	145	1.49 (0.95-2.67)	2.08 (1.04-1.45)*

Note: * = P < 0.05, ** = P < 0.01, *** = P <0.001 NA: not applicable

**¶** ^**=**^adjusted (from multivariate logistic regression model) for socio-economic and demographic characters of parents and children and health caring practices and environmental conditions (detail list available in the data analysis section), but only values of variables which showed significant association either in the bivariate or multivariate regression analysis were reported in the table.

## Discussion

About 31% of the school age children living in *Woreda* 8, Lideta Sub-city, Addis Ababa in March 2014 were undernourished, among which 19.6% were stunted and 15.9% were underweight. Being male, born to a less than 20 years old mother, feeding at most 3 times a day, living in a mud floored house, higher (>2) birth order, having large family size (6–8) and having a protestant or catholic mother were associated with undernutrition.

Undernutriton, in school age children, was reported in the forms of stunting (11% to 42.7%) and underweight (7.2% to 59.7%) in different parts of Ethiopia [14-18]. A 24% stunting was also reported in Addis Ababa [10]. The discrepancies could result from differences in the study methods and existing undernutrition programs. In addition, socio-economic differences between areas (rural vs. urban, for example) could explain the differences in the prevalence of undernutrition across Ethiopia.

Factors such as age, sex and birth order of the child were significantly associated with an increased risk of undernutrition. Undernutrition was common in children who were male, 10 to 14 years old with birth order greater than two. This is in agreement with the studies in different African countries, including Ethiopia [3,15,19-23]. Feeding practices, care and exposure to infection, which primarily determine the nutritional status of children, vary with age [24,25]. Children of age 10 to 14 years are more active and lose a greater amount of energy. Excess energy loss, together with lack of nutritious food, could make them undernourished. Indeed, it was the underweight children (compared to those that were overweight) who demonstrated a high level of physical activity [26]. This may have important public health implications to countries like Ethiopia which require a strategy to keep the energy balacne. Biological factors, inequalities in resource allocation within households and socio-cultural norms prevailing in the community could be responsible for the variation in the risk of undernutrition between males and females [14,27]. The higher prevalence of undrenutrition in children with higher birth orders (greater than two) compared to other children might be explained by the possibility that most Ethiopian parents give less attention, care and resource to older children when they give birth to new ones.

Children belonging to households with 6–8 members were more undernourished than those belonging to households with less than 6–8 members. Similar findings have been documented in other parts of the world [14,28,29]. A large number of household members could contribute to low levels of child care and dietary intake [30-32]. In line with this, undrenutrition was more common in children who had a meal frequency of at most 3 compared to those who had more than 3 per day. Stunting was more common in children living in houses with mud floor than in those living in the cement/brick/ceramic or wood floor, which is consistent with the study in Kenya [33]. The mud floor could indicate the poor socioeconomic status of the parents; this could contribute to the frequent undernutrition in the group. In addition, the mud floor surfaces could be conducive for the growth of pathogenic microorganisms which make children sick and undernourished [24].

One of the strongest determinants of stunting was the mother’s occupation. Children having employed mothers were at greater risk of stunting than those having housewife mothers. This is similar with studies that reported increased prevalence of undernutrition among children whose mothers work outside of their home [34-36]. Mother’ care plays a major role in child nutrition as she is the closest to the child [37-39]. Thus, mothers who stayed at home could spend more time to care for their children.

Stunting was higher in children born to protestant or catholic mothers than to Orthodox or Muslim mothers. This could possibly result from differences in socioeconomics, exposure to infection and level of education. Further studies are needed to better understand why children with protestant or catholic mothers had a higher risk of undernutrition compared to children of orthodox religious mothers. The higher undernutrition risk in children born to mothers less than 20 years old could be due to low food intake by the mothers during pregnancy [40]. The low nutrient level of mothers, during pregnancy, could affect growth of the fetus and the baby during childhood [40]. In Ethiopia, undernutrition is higher in women 15 to 19 years old compared to women in the older ages [41]. In addition, women of less than 20 years of age are not usually employed and have less or no income to feed their children. Moreover, teenage pregnancy has been associated with adverse birth outcomes, including low birth weight and congenital malformations, that can later affect the nutritional status of the children [42,43].

Parental education was not associated with undernutrition in the current study as previously reported [44-46]. Similarly, prevalence of undernutrition did not differ significantly among households having different incomes. Although educated mothers are aware of child nutrition, they could fail to practice it due to cultural, ethnic and religious reasons [47].

The current study is cross-sectional in design which does not establish causal relationship between undernutrition and the socioeconomic, socio-demographic, environmental and household factors. In addition, respondents could fail to provide correct responses to some of the questions and, hence, might introduce recall bias to the data. The numbers of male and female children were not matched and their pubertal stage was not considered in the study which could also have some effect on the outcome.

## Conclusions

Undernutrition is prevalent among school age children living in Woreda 8, Lideta Sub-City, Addis Ababa, Ethiopia. Age, sex, birth order, daily meal frequency, maternal age and family size were major predictors of undernutrition in children. Employment, religious status of the mothers and floor type of child’s house were also associated with the nutritional status of children. In addition to improving economic status of the community, policy makers should consider making greater provisions for health education regarding child nutrition. Nutritional screening and supplementary feeding programs to undernourished children are vital.
